# Frailty, high-sensitivity C-reactive protein and cardiovascular disease: a nationwide prospective cohort study

**DOI:** 10.1007/s40520-025-02928-6

**Published:** 2025-02-28

**Authors:** Lei Zheng, Jianjun Ye, Xinyang Liao, Jing Li, Qihao Wang, Feng Wang

**Affiliations:** 1 Department of Urology, People’s Hospital of Tibet Autonomous Region, Lhasa, 850000 China; 2 Department of Urology, West China Hospital, Sichuan University, Chengdu, China; 3 Department of Cardiology, People’s Hospital of Tibet Autonomous Region, Lhasa, China

**Keywords:** Frailty, High-sensitivity C-reactive protein, Cardiovascular disease, Mediating effect, Joint association

## Abstract

**Background:**

This study aimed to investigate the complex associations of frailty and high-sensitivity C-reactive protein (hsCRP) with cardiovascular disease (CVD) through a nationwide prospective cohort, while also assessing the mediating associations.

**Methods:**

According to critical criteria, a total of 5239 participants from the China Health and Retirement Longitudinal Study (CHARLS) in 2011 were ultimately enrolled in this study. Frailty was evaluated by the frailty index with 40 items, and CVD was defined as the presence of physician-diagnosed heart disease and/or stroke. A restricted cubic spline model, receiver operating characteristic curves, adjusted Cox proportional hazards regression, interaction analyses and mediation analyses were performed for association exploration.

**Results:**

During a maximum follow-up of 7.0 years, 1204 (23.67%) people developed CVD. Both elevated hsCRP and frailty were significantly associated with CVD incidence. Compared with participants with a healthy status and low hsCRP (< 1.015 mg/L), those with a frailty status and elevated hsCRP had the highest risk of CVD (adjusted HR, 2.97; 95% CI 2.29–3.84), heart disease (adjusted HR, 2.93; 95% CI 2.16–3.96), and stroke (adjusted HR, 4.26; 95% CI 2.81–6.44), which were still robust in the subgroup analysis. Moreover, frailty significantly mediated 19.60% of the associations between hsCRP and CVD.

**Conclusions:**

Combined assessment of frailty and hsCRP levels helps to better stratify the individual risk of CVD. Frailty could partly mediate the associations between hsCRP and CVD incidence.

**Supplementary Information:**

The online version contains supplementary material available at 10.1007/s40520-025-02928-6.

## Introduction

With the aging of the world's population, the burden of cardiovascular disease (CVD) has risen steadily in the past 30 years, with a 92.3% increase in the overall prevalent cases (from 271 to 523 million, 1990–2019) and a 53.7% increase in the overall deaths (from 12.1 million to 18.6 million, 1990–2019) [1], posing a substantial threat to both healthcare systems and the well-being of individuals [2]. As a result, there is an urgent need to understand the epidemiological characteristics or risk factors for CVD and to strengthen the existing strategies to identify high-risk individuals for primary prevention.

Frailty, an aging-related multisystem syndrome characterized by weakened physiologic reserve and increased vulnerability to accidental stress, has emerged as a critical contributor to physical disease in elderly people [3]. Many efforts have been made to determine the role of frailty in CVD [4–6], given the tight association between CVD and age (for example, according to the American Heart Association, the incidence rates of CVD are at least 75% in elderly individuals aged 60–79 years and approximately 90% in octogenarians [7]). He D and his coworkers have successfully clarified the significant influence of changes in frailty status on CVD risk by three prospective cohorts [4], revealing the necessity of delaying frailty progression as well as reversing frailty status in CVD practice. The intrinsic inflammation of the body was reported to be one of the overlapping mechanisms underlying both frailty and CVD [8]. Systemic inflammation is a common pathophysiological mechanism and is partly responsible for age-related frailty [9], inferior physiology [10] and degenerated organic function [11, 12], including subclinical CVD or even clinical CVD. High-sensitivity C-reactive protein (hsCRP) has been proposed to be an effective surrogate for measuring systemic inflammation in clinical practice [13]. A recent study demonstrated the substantial association between hsCRP and frailty, subsequently suggesting that reducing systemic inflammation is essential to develop strategies for frailty prevention in middle-aged and elderly people [9]. However, few studies have investigated the associations among frailty, hsCRP and CVD in the same population, and the mutual and mediating associations among frailty, hsCRP and CVD still remain unclear, given the potential unequal mediating effect between frailty and hsCRP in terms of CVD [9, 14].

Therefore, we utilized data from the China Health and Retirement Longitudinal Study (CHARLS), with the intention of providing inspiration and relevant evidence for clinical CVD risk restratification and precise intervention by exploring and determining the associations among frailty, hsCRP and CVD incidence.

## Methods

### Data sources and study population

The present study was a secondary analysis of CHARLS, a widely recognized national population-based cohort study among the middle aged and the elderly, and all original data in the study are available from CHARLS (https://charls.charlsdata.com/pages/data/111/zh-cn.html) upon reasonable request. The conduction and report of this study were in accordance with the Strengthening the Reporting of Observational Studies in Epidemiology (STROBE) reporting guidelines [15].

The CHARLS cohort was established through a multistage probability sampling process in 2011 (Wave 1), totaling 17,708 participants from 10,257 households across 150 counties/districts and 450 villages/resident committees in China. The baseline survey (Wave 1) was completed via standardized questionnaires administered through personal interviews, which spanned from June 2011 to March 2012, and the follow-up interviews were conducted every two years (Wave 2 in 2013, Wave 3 in 2015, Wave 4 in 2018 and the newest Wave 5 in 2020). In particular, information about blood samples could only be obtained at Wave 1 and Wave 3 (11,847 and 13,420 participants, respectively). The detailed explanations of the sampling methods, anthropometric measures, and covariate definitions (including hypertension and diabetes), have been illustrated in previous studies [16–18] and will not be repeated in the present study.

**Fig. 1:**
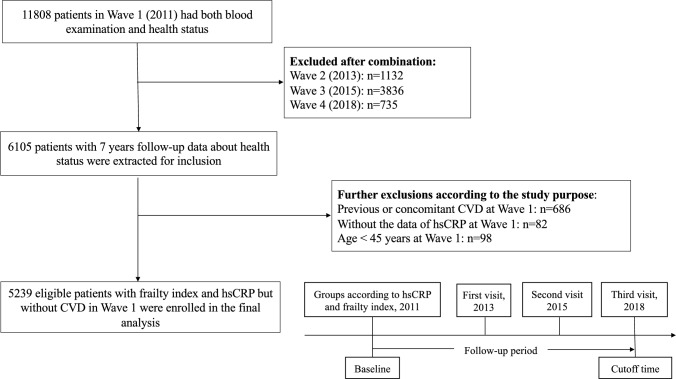
The detailed thoughts and operations on eligible participant selection. *CVD* cardiovascular disease, *hsCRP* high-sensitive C-reactive protein

The valuable data of Wave 5 were not included in our study, and our detailed thoughts and operations on eligible participant selection are depicted in Fig. 1. First, we included 11,808 participants whose blood examination and health status data were collected at Wave 1 (baseline). Then, by using R language software version 3.4.3, we combined the information of Wave 2, Wave 3 and Wave 4 in an orderly manner by the exclusive ID number of each participant on the basis of the selected Wave 1. The eliminant number of participants after each combination was 1132, 3836 and 735 at Wave 2, Wave 3 and Wave 4, respectively. Therefore, 6105 participants with 7-year follow-up data about health status were initially extracted for subsequent analysis. Given the explicit purpose of our study, we further excluded 686 participants who had previous or concomitant CVD at Wave 1, 82 participants without records of hsCRP at Wave 1 and 98 participants aged less than 45 years at baseline. Finally, 5239 participants were eligible for the final analysis of interest.

### Frailty and hsCRP

In this study, we used a continuous variable with values ranging from 0.00 to 1.00, named the frailty index (FI), to evaluate the frailty status of each participant. Following standard procedures [19], the FI in the present study was successfully constructed with 40 items, which were recorded in baseline Wave 1. FI was the accumulation of multiple age-related health deficits, and the evaluated items included previous or concomitant disease (excluding heart disease and stroke), cognition, depression, physical function, and self-assessment (Supplementary Table 1). Except for 6 items with 5 scoring results (scoring 0.00, 0.25, 0.50, 0.75 or 1.00), the remaining 34 items were scored with binary results (scoring 0.00 or 1.00). For each participant, FI was calculated as the sum of the scores of 40 items divided by 40. According to the previous consensus [20], participants were classified into a health group (FI ≤ 0.10), prefrailty group (0.1 < FI < 0.25) and frailty group (FI ≥ 0.25).

The data of hsCRP for each participant came from baseline Wave 1. Given that hsCRP was a continuous variable, we used a restricted cubic spline (RCS) model to examine the shape of the correlation between hsCRP and CVD (Supplementary Fig. 1). A nonlinear association was found (P for nonlinearity < 0.001), and the curve was approximately flat logarithmically shaped. Due to the nonlinear distribution of hsCRP, the data were clarified into two categories: low-level (hsCRP < 1.015 mg/L) and high-level (hsCRP ≥ 1.015 mg/L).

### CVD diagnosis

The diagnostic method for new-onset CVD in this study was in accordance with what had been well described in previous studies [4, 18, 21]. The key items in the questionnaires were as follows: “Have you been diagnosed with stroke/heart disease by a doctor?” and “Are you now undergoing any of the following treatments to treat stroke/heart disease or its complications?”. CVD was defined when new-onset heart disease or stroke was reported during the follow-up period. We will also record the date, which was defined as the previous follow-up period of the interview in which the new-onset CVD was reported. Moreover, for those participants with new-onset CVD, heart disease, stroke and their corresponding dates were separately recorded for subsequent subgroup analysis.

### Covariates

The baseline covariates in this study included age, gender, marital status, educational level, smoking status, drinking status, body mass index (BMI), total cholesterol (TC), high-density lipoprotein cholesterol (HDL-C), low-density lipoprotein cholesterol (LDL-C), glycated hemoglobin (HbA1c), creatinine, total triglyceride (TG), fasting blood glucose (FBG) and hsCRP. Marital status was classified into two categories: married and others (separated, divorced, widowed, never married). Attained education was divided into four levels: no formal education (no formal education, illiterate, sishu, did not finish primary school), primary school, middle or high school, and college or above (vocational school, two/three/four college degrees, postgraduate). Both smoking status and alcohol consumption status were categorized as never or ever (current or former).

### Statistical analysis

The data were analyzed by the R statistical package (http://www.r-projiect.org; version 4.1.3) and Empower (R) (http://www.empowersta ts.com). A two-tailed P < 0.05 was considered to indicate statistical significance.

For the missing information of the above covariates, the multiple imputation method, reported by White and Groenwald [22, 23], was adapted to address missing variables and then to maintain the largest possible sample size. In terms of the baseline characteristics of all participants and groups (hsCRP groups, FI groups, and combination groups), continuous variables were expressed as the means ± standard deviations (SDs) and were compared by one-way ANOVA, while categorical variables were expressed as frequencies with percentages and were compared by Pearson’s chi-squared test.

Receiver operating characteristic (ROC) curves of hsCRP, FI and their combination were generated separately with new-onset CVD-oriented outcome, and the area under the curve (AUC) of each model was compared using the DeLong test to determine the additional predictive value of the combination. Kaplan–Meier curves of the cumulative incidence were used to visually display the differences in the occurrence of outcome events in each group. To determine the associations of FI, hsCRP and their combination with CVD incidence, multivariable-adjusted Cox proportional hazards models were used to calculate hazard ratios (HRs) with 95% confidence intervals (CIs). Three models were included: Model 1 (nonadjusted), Model 2 (adjusted for age, sex, marital status, educational level, smoking status, drinking status, BMI) and Model 3 (adjusted for age, sex, marital status, educational level, smoking status, drinking status, BMI, TC, HDL-C, LDL-C, HbA1c, creatinine, TG and FBG). The effect of FI on CVD incidence stratified by hsCRP was also explored, and vice versa. Furthermore, a variety of subgroup analyses and interaction analyses were performed to assess the robustness of the results.

Given the above results, we sought to explore the mediating role of one factor (FI or hsCRP) in the association between the other factors (hsCRP or FI) and CVD incidence for further understanding the underlying mechanisms involved. Mediation analysis was conducted to evaluate the direct and indirect associations (‘mediation’ package in R software). Briefly, the analysis could be completed by three formulas [24]: (1) Y (outcome, CVD incidence) = βTot X (predictor variable, FI or hsCRP); (2) M (mediator, hsCRP or FI) = β1 X and (3) Y = β2 M + βDir X. In the above formulas, the βTot value represented the total effect, the β1 value represented indirect effect 1, the β2 value represented indirect effect 2, and the βDir value represented the direct effect. Therefore, the mediating effect of the mediator was calculated as (β1 × β2/βTol) × 100%.

## Results

### Baseline characteristics of the study participants

According to the inclusion and exclusion criteria, a total of 5239 participants from the CHARLS were enrolled in this study, with a male to female ratio of approximately 46:54 and a mean age of 58 years. Table 1 shows the characteristics of the participants. The mean hsCRP level at baseline was 2.37 ± 5.78, and the mean FI at baseline was 0.13 ± 0.07.

**Table 1:** Baseline characteristics of included participants

Characteristic	Overall
Participants, n	5239
Age, years, mean (SD)	58.02 (8.95)
Age categorical, n (%)	
< 60	3136 (59.86)
≥ 60	2103 (40.14)
Gender, n (%)	
Female	2821 (53.85)
Male	2418 (46.15)
Marriage, married, n (%)	
Yes	4775 (91.14)
No	464 (8.86)
Educational level, n (%)	
No formal education	2313 (44.15)
Primary school	1193 (22.77)
Middle or high school	1581 (30.18)
College or above	152 (2.90)
Smoking status, n (%)	
Never	3203 (61.14)
Former	445 (8.49)
Current	1591 (30.37)
Drinking status, n (%)	
Never	3859 (73.66)
Former	430 (8.20)
Current	950 (18.14)
Total cholesterol, mean (SD)	193.05 (38.59)
HDL cholesterol, mean (SD)	50.96 (15.45)
LDL cholesterol, mean (SD)	116.63 (34.88)
HbA1c, mean (SD)	5.24 (0.79)
Creatinine, mean (SD)	0.77 (0.18)
Total triglyceride, mean (SD)	134.43 (107.79)
Glucose, mean (SD)	110.11 (34.78)
hsCRP, mean (SD)	2.37 (5.78)
FI, mean (SD)	0.13 (0.07)

*SD* standard deviation, *HDL* high-density lipoprotein, *LDL* low-density lipoprotein, *hsCRP* high-sensitive C-reactive protein, *FI* frailty index, *HbA1c* glycated hemoglobin

At baseline, 2542 (48.52%) participants were divided into a high-hsCRP group according to the RCS model, 2898 (55.31%) participants were labeled as prefrail, and 379 (7.23%) participants were labeled as frail based on the self-constructed items. After combining the two factors, 1094 (20.89%) participants had neither frailty status nor elevated hsCRP, 1452 (27.72%) participants had both prefrailty status and elevated hsCRP, and 222 (4.24%) participants had both frailty status and elevated hsCRP. The specific characteristics of the groups are provided in Supplementary Tables 1–3. Compared to participants without frailty status and elevated hsCRP, those with frailty status and elevated hsCRP tended to be older, female, and to have poor education and drinking experience. Moreover, the co-distribution of FI and hsCRP stratified by new-onset CVD indicated that participants with CVD were more likely to have higher FI and hsCRP (Supplementary Fig. 2). ROC curves and corresponding AUCs evinced that the combination of FI and hsCRP had the best predictive performance in new-onset disease (Supplementary Fig. 3).

### FI, hsCRP and new-onset CVD

**Fig. 2:**
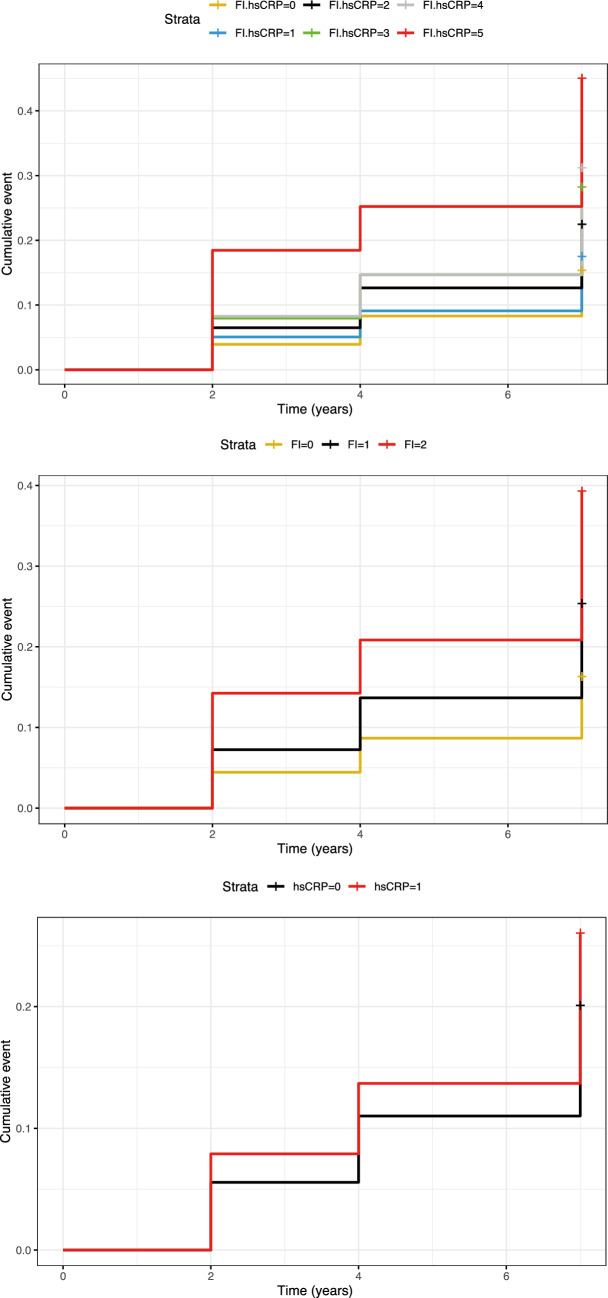
The Kaplan–Meier curves of the cumulative incidence of CVD by frailty index and hsCRP level. *CVD* cardiovascular disease, *hsCRP* high-sensitive C-reactive protein. FI.hsCRP = 0: FI ≤ 0.10 and hsCRP < 1.015 mg/L; FI.hsCRP = 1: FI ≤ 0.10 and hsCRP ≥ 1.015 mg/L; FI.hsCRP = 2: 0.10 < FI < 0.25 and hsCRP < 1.015 mg/L; FI.hsCRP = 3: 0.10 < FI < 0.25 and hsCRP ≥ 1.015 mg/L; FI.hsCRP = 4: FI ≥ 0.25 and hsCRP < 1.015 mg/L; FI.hsCRP = 5: FI ≥ 0.25 and hsCRP ≥ 1.015 mg/L. FI = 0: FI ≤ 0.10; FI = 1: 0.10 < FI < 0.25; FI = 2: FI ≥ 0.25. hsCRP = 0: hsCRP < 1.015 mg/L; hsCRP = 1: hsCRP ≥ 1.015 mg/L

During a maximum follow-up of 7.0 years, 1204 (23.67%) people developed cardiovascular disease, including 886 (16.91%) with heart disease and 446 (8.51%) with stroke. The Kaplan–Meier curves of the cumulative incidence of CVD, heart disease and stroke are shown in Fig. 2 and Supplementary Fig. 4. Supplementary Fig. 5 and Supplementary Fig. 6 present the Kaplan–Meier curves of the cumulative incidence of CVD stratified by FI and hsCRP, respectively. The results of the multivariable-adjusted Cox proportional hazards models are emanated in Table 2. After adjusting potential confounders (in Model 3), compared to participants with health status and low hsCRP, those with frailty status and elevated hsCRP had the highest risk of CVD and were independently associated with increased risk of 197% (HR, 2.97; 95% CI 2.29–3.84), followed by those with frailty status and low hsCRP, those with prefrailty status and elevated hsCRP, those with prefrailty status and low hsCRP and those with heath status and elevated hsCRP. Similar phenomena were observed for heart disease and stroke. The associations between FI or hsCRP and CVD incidence are displayed in Supplementary Table 4.

**Table 2:** Risk of CVD upon co-exposure stratified by the FI and hsCRP

Exposure	Non-adjusted		Adjusted I		Adjusted II	
	HR (95% CI)	P value	HR (95% CI)	P value	HR (95% CI)	P value
*CVD*						
Group 1	Reference		Reference		Reference	
Group 2	1.15 (0.92, 1.43)	0.217	1.14 (0.91, 1.42)	0.247	1.09 (0.87, 1.36)	0.459
Group 3	1.51 (1.25, 1.82)	< 0.001	1.49 (1.23, 1.80)	< 0.001	1.47 (1.22, 1.78)	< 0.001
Group 4	1.92 (1.61, 2.30)	< 0.001	1.85 (1.54, 2.22)	< 0.001	1.75 (1.46, 2.11)	< 0.001
Group 5	2.13 (1.55, 2.92)	< 0.001	2.06 (1.49, 2.84)	< 0.001	2.04 (1.48, 2.82)	< 0.001
Group 6	3.35 (2.62, 4.29)	< 0.001	3.15 (2.44, 4.06)	< 0.001	2.97 (2.29, 3.84)	< 0.001
*Heart disease*						
Group 1	Reference		Reference		Reference	
Group 2	1.04 (0.80, 1.36)	0.760	1.05 (0.81, 1.36)	0.729	1.00 (0.77, 1.31)	0.987
Group 3	1.55 (1.25, 1.93)	< 0.001	1.54 (1.24, 1.92)	< 0.001	1.53 (1.23, 1.91)	< 0.001
Group 4	1.98 (1.60, 2.43)	< 0.001	1.93 (1.56, 2.39)	< 0.001	1.84 (1.48, 2.28)	< 0.001
Group 5	2.06 (1.42, 3.01)	< 0.001	2.03 (1.39, 2.97)	< 0.001	2.01 (1.37, 2.94)	< 0.001
Group 6	3.21 (2.40, 4.30)	< 0.001	3.10 (2.29, 4.18)	< 0.001	2.93 (2.16, 3.96)	< 0.001
*Stroke*						
Group 1	Reference		Reference		Reference	
Group 2	1.54 (1.07, 2.22)	0.022	1.50 (1.04, 2.16)	0.031	1.38 (0.95, 2.00)	0.090
Group 3	1.69 (1.21, 2.34)	0.002	1.67 (1.20, 2.33)	0.002	1.63 (1.17, 2.27)	0.004
Group 4	2.36 (1.72, 3.23)	< 0.001	2.22 (1.62, 3.06)	< 0.001	2.04 (1.48, 2.82)	< 0.001
Group 5	2.54 (1.48, 4.34)	< 0.001	2.45 (1.43, 4.23)	0.001	2.40 (1.40, 4.14)	< 0.001
Group 6	5.05 (3.39, 7.51)	< 0.001	4.72 (3.14, 7.11)	< 0.001	4.26 (2.81, 6.44)	< 0.001

Adjusted Model I: age, sex, marital status, educational level, smoking status, drinking status and body mass index were adjusted; Adjusted Model 2: age, sex, marital status, educational level, smoking status, drinking status, body mass index, total cholesterol, high-density lipoprotein cholesterol, low-density lipoprotein cholesterol, glycated hemoglobin, creatinine, creatinine, total triglyceride and fasting blood glucose were adjusted. Group 1: FI ≤ 0.10 and hsCRP < 1.015 mg/L; Group 2: FI ≤ 0.10 and hsCRP ≥ 1.015 mg/L; Group 3: 0.10 < FI < 0.25 and hsCRP < 1.015 mg/L; Group 4: 0.10 < FI < 0.25 and hsCRP ≥ 1.015 mg/L; Group 5: FI ≥ 0.25 and hsCRP < 1.015 mg/L; Group 6: FI ≥ 0.25 and hsCRP ≥ 1.015 mg/L

*CVD* cardiovascular disease, *hsCRP* high-sensitive C-reactive protein, *FI* frailty index, *HR* hazard ratios, *CI* confidence interval

Subgroup analyses of FI and hsCRP were performed separately to further explore the risk re-stratification of CVD based on FI and hsCRP (Table 3). After adjusting potential confounders (in Model 3), participants with worse health status had a significantly increased risk of CVD, heart disease and stroke events, which was independent of the hsCRP value; whereas the effect of elevated hsCRP on CVD, heart disease and stroke events was nonsignificant in each FI subgroup analysis.

**Table 3:** Risk reclassification of CVD based on the FI and hsCRP

Exposure	CVD		Heart disease		Stroke	
	HR (95% CI)	P value	HR (95% CI)	P value	HR (95% CI)	P value
**Scenario 1**						
FI ≤ 0.10						
hsCRP < 1.015	Reference		Reference		Reference	
hsCRP ≥ 1.015	1.09 (0.87, 1.37)	0.444	1.04 (0.79, 1.36)	0.790	1.30 (0.89, 1.91)	0.178
0.10 < FI < 0.25						
hsCRP < 1.015	Reference		Reference		Reference	
hsCRP ≥ 1.015	1.19 (1.02, 1.38)	0.027	1.19 (1.00, 1.41)	0.054	1.27 (0.99, 1.63)	0.058
FI ≥ 0.25						
hsCRP < 1.015	Reference		Reference		Reference	
hsCRP ≥ 1.015	1.48 (1.03, 2.16)	0.033	1.48 (0.97, 2.27)	0.072	1.66 (0.94, 2.94)	0.084
**Scenario 2**						
hsCRP < 1.015						
FI ≤ 0.10	Reference		Reference		Reference	
0.10 < FI < 0.25	1.47 (1.19, 1.74)	< 0.001	1.51 (1.21, 1.89)	< 0.001	1.53 (1.09, 2.14)	0.014
FI ≥ 0.25	1.93 (1.40, 2.69)	< 0.001	1.97 (1.34, 2.90)	< 0.001	2.14 (1.23, 3.73)	0.007
hsCRP ≥ 1.015						
FI ≤ 0.10	Reference		Reference		Reference	
0.10 < FI < 0.25	1.66 (1.38, 2.01)	< 0.001	1.87 (1.49, 2.35)	< 0.001	1.55 (1.15, 2.09)	0.004
FI ≥ 0.25	2.88 (2.21, 3.75)	< 0.001	2.98 (2.17, 4.10)	< 0.001	3.41 (2.29, 5.09)	< 0.001

This analysis was implemented in the adjusted Model 2: age, sex, marital status, educational level, smoking status, drinking status, body mass index, total cholesterol, high-density lipoprotein cholesterol, low-density lipoprotein cholesterol, glycated hemoglobin, creatinine, creatinine, total triglyceride and fasting blood glucose were adjusted. Scenario 1: effect of hsCRP on CVD between FI groups; scenario 2: effect of FI on CVD between hsCRP groups

*CVD* cardiovascular disease, *hsCRP* high-sensitive C-reactive protein, *FI* frailty index, *HR* hazard ratios, *CI* confidence interval

### Subgroup analysis and interaction analyses

A series of subgroup analyses were conducted to further investigate the association between combination of FI and hsCRP and CVD incidence. As shown in Table 4, none of the subgroups, including age, gender, marital status, educational level, smoking status, drinking status, TC, HDL-C, LDL-C, HbA1c, creatinine, TG, and FBG, significantly changed the association between combination of FI and hsCRP and CVD incidence (all P values for interaction > 0.05). The subgroup analyses on incidence of heart disease and stroke are provided in Supplementary Table 5 and Supplementary Table 6.

**Table 4:** The subgroup analyses and interaction analyses about the risk of CVD upon co-exposure stratified by the FI and hsCRP

Subgroup	Group 1 (n = 1094)	Group 2 (n = 868)	Group 3 (n = 1446)	Group 4 (n = 1452)	Group 5 (n = 157)	Group 6 (n = 222)	P for interaction
	HR (95% CI)	HR (95% CI)	HR (95% CI)	HR (95% CI)	HR (95% CI)	HR (95% CI)	
Age							0.433
< 60	Reference	1.16 (0.88, 1.53)	1.45 (1.13, 1.85)	1.93 (1.52, 2.45)	2.45 (1.55, 3.88)	3.71 (2.56, 5.49)	
≥ 60	Reference	1.77 (1.10, 2.85)	2.63 (1.73, 3.98)	2.83 (1.89, 4.27)	3.07 (1.80, 5.25)	4.55 (2.90, 7.17)	
Gender							0.834
Female	Reference	1.09 (0.80, 1.50)	1.59 (1.26, 2.05)	1.83 (1.45, 2.37)	2.28 (1.56, 3.39)	2.97 (2.10, 4.19)	
Male	Reference	1.34 (0.80, 2.25)	1.65 (1.01, 2.69)	2.07 (1.27, 3.38)	2.11 (1.04, 4.30)	3.87 (2.23, 6.70)	
Married							0.225
No	Reference	1.05 (0.83, 1.36)	1.44 (1.19, 1.76)	1.76 (1.45, 2.14)	2.17 (1.54, 3.05)	3.20 (2.45, 4.17)	
Yes	Reference	1.39 (0.57, 3.40)	1.63 (0.77, 3.47)	1.70 (0.80, 3.58)	1.15 (0.39, 3.46)	1.61 (0.59, 4.40)	
Smoking status							0.752
Never	Reference	1.06 (0.79, 1.43)	1.58 (1.24, 2.01)	1.84 (1.44, 2.35)	2.20 (1.48, 3.27)	2.95 (2.10, 4.13)	
Current/former	Reference	1.97 (1.16, 3.37)	2.31 (1.40, 3.81)	2.85 (1.74, 4.68)	2.99 (1.51, 5.91)	5.43 (3.12, 9.46)	
Drinking status							0.197
Never	Reference	0.97 (0.74, 1.27)	1.43 (1.14, 1.78)	1.68 (1.35, 2.09)	2.10 (1.49, 2.99)	2.68 (2.01, 3.57)	
Current/former	Reference	1.45 (0.80, 2.63)	1.60 (0.92, 2.81)	2.02 (1.16, 3.55)	1.20 (0.40, 3.61)	5.16 (2.42, 10.85)	
Dyslipidaemia							0.546
No	Reference	1.10 (0.80, 1.52)	1.312 (1.00, 1.76)	1.86 (1.40, 2.39)	1.96 (1.24, 3.11)	2.74 (1.86, 4.04)	
Yes	Reference	1.47 (0.88, 2.45)	2.190 (1.36, 3.52)	2.30 (1.42, 3.71)	2.82 (1.55, 5.13)	4.31 (2.54, 7.30)	
Hypertension							0.660
No	Reference	1.11 (0.79, 1.56)	1.58 (1.20, 2.08)	1.77 (1.34, 2.34)	1.85 (1.07, 3.18)	3.66 (2.51, 5.35)	
Yes	Reference	1.30 (0.82, 2.04)	1.70 (1.11, 2.61)	2.10 (1.38, 3.22)	2.56 (1.51, 4.32)	3.11 (1.91, 5.06)	
Kidney disease							1.000
No	Reference	1.06 (0.76, 1.47)	1.42 (1.09, 1.85)	1.68 (1.30, 2.18)	1.96 (1.26, 3.05)	2.87 (2.01, 4.11)	
Yes	Reference	1.24 (0.77, 1.99)	1.66 (1.07, 2.58)	2.01 (1.30, 3.11)	2.32 (1.30, 4.15)	3.40 (2.06, 5.63)	
Diabetes							0.867
No	Reference	1.21 (0.88, 1.66)	1.63 (1.26, 2.12)	1.92 (1.47, 2.51)	2.25 (1.42, 3.56)	3.08 (2.10, 4.54)	
Yes	Reference	1.27 (0.80, 2.02)	1.69 (1.09, 2.60)	2.06 (1.34, 3.17)	2.40 (1.37, 4.19)	3.73 (2.29, 6.08)	

This analysis was implemented in the adjusted Model 2: age, sex, marital status, educational level, smoking status, drinking status, body mass index, total cholesterol, high-density lipoprotein cholesterol, low-density lipoprotein cholesterol, glycated hemoglobin, creatinine, creatinine, total triglyceride and fasting blood glucose were adjusted. Group 1: FI ≤ 0.10 and hsCRP < 1.015 mg/L; Group 2: FI ≤ 0.10 and hsCRP ≥ 1.015 mg/L; Group 3: 0.10 < FI < 0.25 and hsCRP < 1.015 mg/L; Group 4: 0.10 < FI < 0.25 and hsCRP ≥ 1.015 mg/L; Group 5: FI ≥ 0.25 and hsCRP < 1.015 mg/L; Group 6: FI ≥ 0.25 and hsCRP ≥ 1.015 mg/L

*CVD* cardiovascular disease, *hsCRP* high-sensitive C-reactive protein, *FI* frailty index, *HR* hazard ratios, *CI* confidence interval

### Exploration of mediating effects

**Fig. 3:**
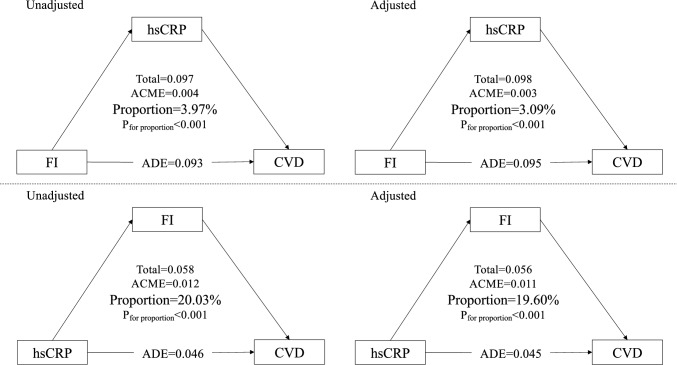
Mutual mediation effects of the hsCRP and frailty index on CVD. *CVD* cardiovascular disease, *hsCRP* high-sensitive C-reactive protein, *FI* frailty index. Adjusted Model: age, sex, marital status, educational level, smoking status, drinking status, body mass index, total cholesterol, high-density lipoprotein cholesterol, low-density lipoprotein cholesterol, glycated hemoglobin, creatinine, creatinine, total triglyceride and fasting blood glucose were adjusted

The mutual mediating effects of frailty and hsCRP on CVD incidence are summarized in Fig. 3. Frailty played a significant mediating role in the association between hsCRP and CVD events, with a quantitative value of 19.60% (P < 0.001), while hsCRP also had a significant mediating effect on the association between FI and CVD incidence, only with a quantitative value of 3.09% (P < 0.001). The percentages of patients who experienced heart disease were 19.01% (P = 0.020) and 2.58% (P < 0.001), respectively (Supplementary Fig. 7). The percentages of patients who experienced stroke were 14.99% (P = 0.020) and 4.4% (P < 0.001), respectively (Supplementary Fig. 8).

## Discussion

Based on a nationwide prospective cohort study, we successfully determined the associations between baseline frailty status, hsCRP and CVD incidence. Participants with worse health status (frailty or prefrailty) and elevated hsCRP significantly tended to have increased CVD risks, and those with frailty status and elevated hsCRP had the highest risk of incident CVD. The associations remained robust even after adjusting for potential confounders. Moreover, our study demonstrated that frailty status partially mediated the association between hsCRP and CVD incidence.

With the explosive growth of the aging population, more attention has been given to the field of frailty, and the original concept of frailty has gradually evolved to a more comprehensive and accurate level. Positive associations between prefrailty or frailty and incident CVD have also been reported in previous studies. By retrospectively analyzing more than 3 million American veterans, Shrauner and his coworkers found that frailty was significantly associated with an increased risk of myocardial infarction and stroke, and the more severe the frailty was, the greater the risk was [25]. Moreover, the tight correlation between the presence and severity of frailty and CVD mortality was also emphasized in that study. According to UK Biobank data collected between 2006 and 2010, Cao et al. reported that individuals with frailty, even in the very early stage of frailty, have a significantly increased risk of incident CVD [26]. Conversely, CVD is also a risk factor for frailty owing to the characteristics of multisystem syndrome. CVD affects multiple aspects of individuals, such as cognition and physical function, which directly leads to a tendency toward high frailty scores. The reported frailty prevalence ranges from 36.2 to 52% among patients with heart failure [27], from 26 to 68% among those undergoing aortic valve replacement [28], and approximately 45.4% among those undergoing percutaneous mitral valve repair [29]. Therefore, during CVD treatment, it is highly important to evaluate the frailty status of patients and develop targeted interventions to reduce the degree of frailty and obtain the expected therapeutic effect. The interconnected correlation between frailty and CVD reveals great difficulties in mechanism exploration. Additional studies are warranted to elucidate the potential mechanisms by which frailty and CVD are mutually correlated to help develop more precise clinical guidelines.

A positive association between chronic inflammation and new-onset CVD was also observed in this prospective Chinese cohort. The role of chronic inflammation in CVD progression has been well established [30]. Chronic inflammation is believed to initiate atherothrombosis progression, and hsCRP, the most widely accepted inflammatory marker, has been proposed to accelerate atherosclerosis progression through a variety of potential mechanisms, including facilitating monocyte adhesion and transmigration into the vessel wall [31] and catalyzing M1 macrophage polarization [32]. In a prospective cohort with a total of 22,071 Americans, Ridker et al. reviewed 543 participants who developed myocardial infarction (MI) and ischemic stroke during a follow-up period of more than 8 years and reported a close association between MI, ischemic stroke and elevated hsCRP [33]. Interestingly, they also observed an association between aspirin use and a reduced risk of first MI, which was attributed to changes in the CRP level caused by aspirin [33]. Furthermore, hsCRP also plays a nonnegligible role in the relationship between other risk factors and CVD incidence. Zhang and his coworkers reported that concomitant hsCRP elevation is a prerequisite for the risk of lipoprotein-associated atherosclerotic CVD [34]. Based on data from the Utrecht Cardiovascular Cohort-Second Manifestations of ARTerial (UCC-SMART), Burger et al. revealed that CRP was an independent risk factor for incident heart failure in patients with established CVD [35], indicating its predictive value for CVD prognosis. However, in a two-sample bidirectional Mendelian randomization study conducted by Kuppa, no evidence of reverse causation between CVD and CRP was found, but the causal effect turned out to be statistically significant when CVD was limited to hypertensive heart disease [36]. Due to the inherent limitations of the above studies, including the unitary ethnicity of participants and a lack of individual-level data, well-designed studies with primary data are urgently needed to explore the causal effect between hsCRP and CVD incidence and provide strong evidence for clinical prevention and treatment.

An elusive link existed between chronic inflammation and frailty. Previous observational studies have yielded contradictory results regarding the relationship between chronic inflammation and frailty. A meta-analysis conducted by Soysal et al. pooled 35 studies and revealed that frailty and prefrailty were associated with increased CRP levels [14]. Recently, Luo et al. not only found a significant longitudinal association between hsCRP and frailty based on CHARLS data but also revealed a causal effect through genetic analysis [9]. However, three other longitudinal studies failed to find a significant association between hsCRP and frailty [37–39]. Owing to the complexity of hsCRP and frailty, the associations between CVD and the two abovementioned factors were easily affected by definitions and evaluation criteria of frailty, population differences and other confounding factors, emphasizing the necessity to understand the internal interconnection and mechanism involved.

Furthermore, our results showed the superior performance of the combined evaluation of frailty and hsCRP for identifying and stratifying individuals at relatively high CVD risk. Given that hsCRP has been validated as a prominent risk factor for frailty by genetic analysis [9] and the significant association between hsCRP and frailty with CVD, we hypothesized that frailty could mediate the effect of hsCRP on CVD incidence. Subsequently, a mediating effect exploration indicated that frailty potentially mediated approximately 20% of the association between hsCRP and CVD, while hsCRP mediated only 3.09% of the association between frailty and CVD, which was consistent with our initial hypothesis. There are latent mechanisms regulating the mediating effect of frailty on the association between hsCRP and CVD incidence. Long-term chronic inflammation may contribute to muscle atrophy and abnormal physical function, leading to frailty and vice versa; this vicious cycle ultimately causes mobility deterioration, leading to increased CVD risk [40]. Driven by a variety of factors, including oxidative stress, chronic inflammation leads to the accumulation of cell dysfunction, senescence and even damage [41], which can directly induce frailty status and increased CVD risk.

Notably, this study is the first to comprehensively evaluate the joint association of frailty and hsCRP with CVD through a nationwide prospective cohort and to demonstrate that frailty partially mediates the significant association between hsCRP and CVD, providing additional evidence for existing related studies and providing references for future studies and clinical practice. However, the results should be interpreted in the context of limitations. First, the participants ultimately included in the analysis were selected by strict exclusion criteria rather than random methods, which might have led to selection bias and further weakened the extrapolation of our conclusions. Second, frailty was self-reported by questionnaires. Information bias might decrease the robustness of the results, and more objective modalities for frailty assessment in large populations are urgently needed. Third, all interesting findings in this study were entirely based on middle-aged and aged Chinese individuals; therefore, the findings may not be fully generalizable to populations of all ages and other ethnicities. These findings should be strengthened by incorporating other prospective cohorts, such as the English Longitudinal Study of Aging (ELSA) and the Health and Retirement Study (HRS).

## Conclusions

In summary, by analyzing a nationwide prospective cohort of middle-aged and elderly individuals in China, we found that frailty significantly mediated the association between hsCRP and CVD risk. Combined assessment of frailty and hsCRP helps to better stratify the individual risk of CVD. Future studies are needed to explore the underlying mechanisms and validate the significance of our findings in clinical practice.

## Supplementary Information

Below is the link to the electronic supplementary material.

Supplementary file1 (DOCX 2736 KB)

## Data Availability

The datasets used and/or analyzed during the current study are publicly available or from the corresponding author upon reasonable request. All the authors verify that all the information and materials in the manuscript are original.
