# Supplementary material for: Frailty, high-sensitivity C-reactive protein and cardiovascular disease: a nationwide prospective cohort study
Source: Aging Clin Exp Res. 2025 Feb 28;37(1):58. doi: 10.1007/s40520-025-02928-6 (PMC11870887; doi:10.1007/s40520-025-02928-6)
Supplement: Supplementary file 1 — Supplementary file1 (DOCX 2736 KB) [file 40520_2025_2928_MOESM1_ESM.docx]

**Supplementary file captions**

**Supplementary Table 1.** Questionnaire items of frailty index.

**Supplementary Table 2.** Baseline characteristics according to frailty index.

**Supplementary Table 3.** Baseline characteristics according to high sensitive C-reactive protein.

**Supplementary Table 4.** Baseline characteristics according to frailty index and high sensitive C-reactive protein.

**Supplementary Table 5.** Associations of high sensitive C-reactive protein and frailty index with cardiovascular disease incidence, respectivrly.

**Supplementary Table 6.** Associations of different groups of frailty index and high sensitive C-reactive protein with heart disease incidence stratified by different factors.

**Supplementary Table 7**. Associations of different groups of frailty index and high sensitive C-reactive protein with stroke incidence stratified by different factors.

**Supplementary Figure 1.** Nonlinear association between high sensitive C-reactive protein and cardiovascular disease.

**Supplementary Figure 2.** The codistribution of the frailty index and high sensitive C-reactive protein stratified by incident cardiovascular disease.

**Supplementary Figure 3.** The Receiver operating characteristic curves of high sensitive C-reactive protein, frailty index and their combination about new-onset cardiovascular disease.

**Supplementary Figure 4.** The Kaplan–Meier curves of the cumulative incidence of heart disease and stroke.

**Supplementary Figure 5.** The Kaplan–Meier curves of the cumulative incidence of cardiovascular disease stratified by high frailty index.

**Supplementary Figure 6.** The Kaplan–Meier curves of the cumulative incidence of cardiovascular disease stratified by sensitive C-reactive protern.

**Supplementary Figure 7.** Mutual mediation effects of the high sensitive C-reactive protein and frailty index on heart disease.

**Supplementary Figure 8.** Mutual mediation effects of the high sensitive C-reactive protein and frailty index on stroke.

**Supplementary Table 1.** Questionnaire Items of Frailty Index

| **Definition** | **Coding of variables** |
| --- | --- |
| Self-reported diagnosis of hypertension by a doctor, self-reported use of antihypertension drugs, systolic blood pressure measured to be ≥ 140mmHg, or Diastolic blood pressure measured to be ≥ 90mmHg | Yes=1.00; no=0.00 |
| Self-reported diagnosis of dyslipidemia by a doctor | Yes=1.00; no=0.00 |
| Self-reported diagnosis of chronic lung diseases by a doctor | Yes=1.00; no=0.00 |
| Self-reported diagnosis of liver diseases by a doctor | Yes=1.00; no=0.00 |
| Self-reported diagnosis of stomach or other digestive diseases by a doctor | Yes=1.00; no=0.00 |
| Self-reported diagnosis of emotional, nervous, or psychiatric problems by a doctor | Yes=1.00; no=0.00 |
| Self-reported diagnosis of asthma by a doctor | Yes=1.00; no=0.00 |
| Self-reported diagnosis of memory-related disease by a doctor | Yes=1.00; no=0.00 |
| Self-reported diagnosis of diabetes | Yes=1.00; no=0.00 |
| Self-reported diagnosis of cancer by a doctor | Yes=1.00; no=0.00 |
| Self-reported diagnosis of chronic kidney disease by a doctor | Yes=1.00; no=0.00 |
| Self-reported diagnosis of Arthritis or Rheumatism by a doctor | Yes=1.00; no=0.00 |
| Cognitive impairments (MMSE) | <10,1.00; 11-17,0.75; 18-20,0.50;21-24,0.25;>=25,0.00； |
| Depressive symptoms (the CES-D scale) | >=12,1.00; otherwise,0.00 |
| Do you have any difficulty with running or jogging about 1Km? | Yes, I have difficulty and need help, or I can not do it,1.00; otherwise,0.00 |
| Do you have difficulty with walking 1 km? | Yes, I have difficulty and need help, or I can not do it,1.00; otherwise,0.00 |
| Do you have difficulty with walking 100 metres? | Yes, I have difficulty and need help, or I can not do it,1.00; otherwise,0.00 |
| Do you have difficulty with getting up from a chair. After sitting for along period? | Yes, I have difficulty and need help, or I can not do it,1.00; otherwise,0.00 |
| Do you have difficulty with climbing several flights of stairs without resting? | Yes, I have difficulty and need help, or I can not do it,1.00; otherwise,0.00 |
| Do you have difficulty with stooping, kneeling, or crouching? | Yes, I have difficulty and need help, or I can not do it,1.00; otherwise,0.00 |
| Do you have difficulty with reaching or extending your arms above shoulder level? | Yes, I have difficulty and need help, or I can not do it,1.00; otherwise,0.00 |
| Do you have difficulty with lifting or carrying weights over10 jin? | Yes, I have difficulty and need help, or I can not do it,1.00; otherwise,0.00 |
| Do you have difficulty with picking up a small coin from a table? | Yes, I have difficulty and need help, or I can not do it,1.00; otherwise,0.00 |
| Because of health and memory problems, do you have any difficulty with dressing? | Yes, I have difficulty and need help, or I can not do it,1.00; otherwise,0.00 |
| Do you have any difficulty with bathing or showering? | Yes, I have difficulty and need help, or I can not do it,1.00; otherwise,0.00 |
| Do you have any difficulty with eating, such as cutting Up your food? | Yes, I have difficulty and need help, or I can not do it,1.00; otherwise,0.00 |
| Do you have any difficulty with getting into or out of bed? | Yes, I have difficulty and need help, or I can not do it,1.00; otherwise,0.00 |
| Do you have any difficulties with using the toilet, Including getting up and down? | Yes, I have difficulty and need help, or I can not do it,1.00; otherwise,0.00 |
| Do you have any difficulties with controlling urination and defecation? | Yes, I have difficulty and need help, or I can not do it,1.00; otherwise,0.00 |
| Do you have any difficulties with doing household chores? | Yes, I have difficulty and need help, or I can not do it,1.00; otherwise,0.00 |
| Do you have any difficulties with preparing hot meals? | Yes, I have difficulty and need help, or I can not do it,1.00; otherwise,0.00 |
| Do you have any difficulties with shopping for groceries? | Yes, I have difficulty and need help, or I can not do it,1.00; otherwise,0.00 |
| Do you have any difficulties with managing your money | Yes, I have difficulty and need help, or I can not do it,1.00; otherwise,0.00 |
| Do you have any difficulties with taking medications? | Yes, I have difficulty and need help, or I can not do it,1.00; otherwise,0.00 |
| Do you have one of physical or brain damage/intellectual disabilities? | Yes=1.00; no=0.00 |
| Is your hearing very good, good, fair, poor, or very poor? | Very poor,1.00; poor,0.75; fair,0.5; good,0.25; very good,0 |
| How good is your eyesight for seeing things at a distance? | Very poor,1.00; poor,0.75; fair,0.5; good,0.25; very good,0 |
| How good is your eyesight for seeing things up close? | Very poor,1.00; poor,0.75; fair,0.5; good,0.25; very good,0 |
| Would you say your health is very good, good, fair, poor or very poor? | Very poor,1.00; poor,0.75; fair,0.5; good,0.25; very good,0 |
| Think about your life-as-a-whole. How sat is fie dare you with it? | Very poor,1.00; poor,0.75; fair,0.5; good,0.25; very good,0 |

**Supplementary Table 2.** Baseline characteristics according to frailty index.

| Characteristic | Health group | Pre-frailty group | Frailty group | P value |
| --- | --- | --- | --- | --- |
| Participants, no | 1962 | 2898 | 379 |  |
| Age, years, mean(SD) | 55.83 (8.53) | 58.89 (8.76) | 62.70 (9.58) | <0.001 |
| Age categorical, n (%) |  | | | <0.001 |
| <60 | 1381 (70.39) | 1608 (55.49) | 147 (38.79) |  |
| >=60 | 581 (29.61) | 1290 (44.51) | 232 (61.21) |  |
| Gender, n (%) |  | | | <0.001 |
| Female | 956 (48.73) | 1625 (56.07) | 240 (63.32) |  |
| Male | 1006 (51.27) | 1273 (43.93) | 139 (36.68) |  |
| Marriage, married, n (%) |  | | | <0.001 |
| Yes | 1827 (93.12) | 2618 (90.34) | 330 (87.07) |  |
| No | 135 (6.88) | 280 (9.66) | 49 (12.93) |  |
| Educational level, n (%) |  | | | <0.001 |
| No formal education | 656 (33.44) | 1402 (48.38) | 255 (67.28) |  |
| Primary school | 445 (22.68) | 678 (23.40) | 70 (18.47) |  |
| Middle or high school | 781 (39.81) | 749 (25.85) | 51 (13.46) |  |
| College or above | 80 (4.08) | 69 (2.38) | 3 (0.79) |  |
| Smoking status, n (%) |  | | | 0.007 |
| Never | 1147 (58.46) | 1812 (62.53) | 244 (64.38) |  |
| Current/former | 815 (41.54) | 1086 (37.47) | 135 (35.62) |  |
| Drinking status, n (%) |  | | | <0.001 |
| Never | 1353 (68.96) | 2177 (75.12) | 329 (86.81) |  |
| Current/former | 609 (31.04) | 721 (24.88) | 50 (13.19) |  |
| Total cholesterol, mean(SD) | 191.91 (37.34) | 193.76 (39.39) | 193.46 (38.74) | 0.442 |
| HDL cholesterol, mean(SD) | 51.51 (15.41) | 50.79 (15.50) | 49.41 (15.21) | 0.028 |
| LDL cholesterol, mean(SD) | 115.94 (34.38) | 116.93 (35.41) | 117.87 (33.29) | 0.605 |
| HbA1c, mean(SD) | 5.17 (0.70) | 5.27 (0.82) | 5.34 (0.95) | <0.001 |
| Creatinine, mean(SD) | 0.77 (0.18) | 0.76 (0.18) | 0.75 (0.20) | 0.006 |
| Total triglyceride, mean(SD) | 130.52 (99.96) | 136.73 (112.90) | 137.05 (106.51) | 0.008 |
| Glucose, mean(SD) | 107.48 (29.33) | 111.12 (36.28) | 115.93 (46.18) | <0.001 |
| hsCRP, mean(SD) | 2.02 (5.05) | 2.50 (6.10) | 3.23 (6.58) | <0.001 |

^a^Abbreviations: SD, standard deviation; HDL, high-density lipoprotein; LDL, low-density lipoprotein; hsCRP, high-sensitive C-reactive protein; HbA1c, glycated hemoglobin.

**Supplementary Table 3.** Baseline characteristics according to high sensitive C-reactive protein.

| Characteristic | hsCRP < 1.015 | hsCRP ≥ 1.015 | P-value |
| --- | --- | --- | --- |
| Participants, no | 2697 | 2542 |  |
| Age, years, mean(SD) | 57.10 (8.86) | 58.99 (8.94) | <0.001 |
| Age categorical, n (%) |  |  |  |
| <60 | 1734 (64.29) | 1402 (55.15) |  |
| >=60 | 963 (35.71) | 1140 (44.85) |  |
| Gender, n (%) |  |  | 0.088 |
| Female | 1483 (54.99) | 1338 (52.64) |  |
| Male | 1214 (45.01) | 1204 (47.36) |  |
| Marriage, married, n (%) |  |  | 0.004 |
| Yes | 2488 (92.25) | 2287 (89.97) |  |
| No | 209 (7.75) | 255 (10.03) |  |
| Educational level, n (%) |  |  | 0.021 |
| No formal education | 1162 (43.08) | 1151 (45.28) |  |
| Primary school | 618 (22.91) | 575 (22.62) |  |
| Middle or high school | 852 (31.59) | 729 (28.68) |  |
| College or above | 65 (2.41) | 87 (3.42) |  |
| Smoking status, n (%) |  |  | 0.031 |
| Never | 1687 (62.55) | 1516 (59.64) |  |
| Current/former | 1010 (37.45) | 1026 (40.36) |  |
| Drinking status, n (%) |  |  | 0.929 |
| Never | 1988 (73.71) | 1871 (73.60) |  |
| Current/former | 709 (26.29) | 671 (26.40) |  |
| Total cholesterol, mean(SD) | 190.54 (37.63) | 195.71 (39.42) | <0.001 |
| HDL cholesterol, mean(SD) | 53.56 (15.78) | 48.20 (14.61) | <0.001 |
| LDL cholesterol, mean(SD) | 114.66 (33.22) | 118.71 (36.44) | <0.001 |
| HbA1c, mean(SD) | 5.15 (0.66) | 5.33 (0.89) | <0.001 |
| Creatinine, mean(SD) | 0.76 (0.18) | 0.78 (0.19) | <0.001 |
| Total triglyceride, mean(SD) | 122.59 (97.41) | 146.99 (116.51) | <0.001 |
| Glucose, mean(SD) | 106.44 (27.75) | 114.00 (40.58) | <0.001 |
| hsCRP, mean(SD) | 0.56 (0.22) | 4.30 (7.84) 2.01 | <0.001 |

^a^Abbreviations: SD, standard deviation; HDL, high-density lipoprotein; LDL, low-density lipoprotein; FI, frailty index; HbA1c, glycated hemoglobin.

**Supplementary Table 4.** Baseline characteristics according to frailty index and high sensitive C-reactive protein.

| Characteristic | Group 1 | Group 2 | Group 3 | Group 4 | Group 5 | Group 6 | P-value |
| --- | --- | --- | --- | --- | --- | --- | --- |
| Participants, no | 1094 | 868 | 1446 | 1452 | 157 | 222 |  |
| Age, years, mean(SD) | 55.28 (8.64) | 56.52 (8.33) | 57.97 (8.64) | 59.81 (8.78) | 61.90 (9.46) | 63.26 (9.64) | <0.001 |
| Age categorical, n (%) |  |  |  |  |  |  | <0.001 |
| <60 | 3136 (59.86) | 798 (72.94) | 583 (67.17) | 865 (59.82) | 71 (45.22) | 146 (65.77) |  |
| >=60 | 2103 (40.14) | 296 (27.06) | 285 (32.83) | 581 (40.18) | 86 (54.78) | 2103 (40.14) |  |
| Gender, n (%) |  |  |  |  |  |  | <0.001 |
| Female | 555 (50.73) | 401 (46.20) | 824 (56.98) | 801 (55.17) | 104 (66.24) | 136 (61.26) |  |
| Male | 539 (49.27) | 467 (53.80) | 622 (43.02) | 651 (44.83) | 53 (33.76) | 86 (38.74) |  |
| Marriage, married, n (%) |  |  |  |  |  |  | <0.001 |
| Yes | 1023 (93.51) | 804 (92.63) | 1331 (92.05) | 1287 (88.64) | 134 (85.35) | 196 (88.29) |  |
| No | 71 (6.49) | 64 (7.37) | 115 (7.95) | 165 (11.36) | 23 (14.65) | 26 (11.71) |  |
| Educational level, n (%) |  |  |  |  |  |  | <0.001 |
| No formal education | 352 (32.18) | 304 (35.02) | 698 (48.27) | 704 (48.48) | 112 (71.34) | 143 (64.41) |  |
| Primary school | 259 (23.67) | 186 (21.43) | 332 (22.96) | 346 (23.83) | 27 (17.20) | 43 (19.37) |  |
| Middle or high school | 446 (40.77) | 335 (38.59) | 388 (26.83) | 361 (24.86) | 18 (11.46) | 33 (14.86) |  |
| College or above | 37 (3.38) | 43 (4.95) | 28 (1.94) | 41 (2.82) | 0 (0.00) | 3 (1.35) |  |
| Smoking status, n (%) |  |  |  |  |  |  | 0.006 |
| Never | 649 (59.32) | 498 (57.37) | 932 (64.45) | 880 (60.61) | 106 (67.52) | 138 (62.16) |  |
| Current/former | 445 (40.68) | 370 (42.63) | 514 (35.55) | 572 (39.39) | 51 (32.48) | 84 (37.84) |  |
| Drinking status, n (%) |  |  |  |  |  |  | <0.001 |
| Never | 756 (69.10) | 597 (68.78) | 1100 (76.07) | 1077 (74.17) | 132 (84.08) | 197 (88.74) |  |
| Current/former | 338 (30.90) | 271 (31.22) | 346 (23.93) | 375 (25.83) | 25 (15.92) | 25 (11.26) |  |
| Total cholesterol, mean(SD) | 190.20 (35.39) | 194.06 (39.57) | 190.84 (39.35) | 196.68 (39.22) | 190.17 (36.72) | 195.78 (40.02) | <0.001 |
| HDL cholesterol, mean(SD) | 53.99 (15.46) | 48.38 (14.78) | 53.30 (16.06) | 48.28 (14.49) | 52.89 (15.25) | 46.95 (14.72) | <0.001 |
| LDL cholesterol, mean(SD) | 115.24 (32.73) | 116.83 (36.35) | 114.09 (33.73) | 119.76 (36.80) | 116.01 (31.93) | 119.18 (34.24) | <0.001 |
| HbA1c, mean(SD) | 5.09 (0.61) | 5.27 (0.79) | 5.19 (0.70) | 5.34 (0.92) | 5.19 (0.65) | 5.45 (1.10) | <0.001 |
| Creatinine, mean(SD) | 0.76 (0.17) | 0.80 (0.19) | 0.76 (0.18) | 0.77 (0.18) | 0.73 (0.15) | 0.77 (0.23) | <0.001 |
| Total triglyceride, mean(SD) | 118.27 (86.31) | 145.96 (113.05) | 126.17 (107.24) | 147.25 (117.36) | 119.69 (70.82) | 149.33 (124.48) | <0.001 |
| Glucose, mean(SD) | 104.81 (27.44) | 110.85 (31.24) | 107.43 (27.99) | 114.80 (42.68) | 108.69 (27.34) | 121.05 (55.29) | <0.001 |
| hsCRP, mean(SD) | 0.55 (0.22) | 3.86 (7.17) | 0.56 (0.22) | 4.43 (8.17) | 0.58 (0.22) | 5.11 (8.10) | <0.001 |

^a^Abbreviations: SD, standard deviation; HDL, high-density lipoprotein; LDL, low-density lipoprotein; HbA1c, glycated hemoglobin

**Supplementary Table 5.** Associations of hsCRP and FI with CVD incidence, respectivrly.

| Exposure | Non-adjusted | | Adjust I | | Adjust II | |
| --- | --- | --- | --- | --- | --- | --- |
|  | HR（95% CI） | P value | HR（95% CI） | P value | HR（95% CI） | P value |
| Scenario 1 | | | | | | |
| CVD | | | | | | |
| hsCRP < 1.015 | Reference | | Reference | | Reference | |
| hsCRP ≥ 1.015 | 1.32 (1.18, 1.48) | <0.001 | 1.28 (1.14, 1.44) | <0.001 | 1.22 (1.08, 1.37) | <0.001 |
| Heart disease | | | | | | |
| hsCRP < 1.015 | Reference | | Reference | | Reference | |
| hsCRP ≥ 1.015 | 1.29 (1.13, 1.47) | <0.001 | 1.26 (1.11, 1.44) | <0.001 | 1.20 (1.05, 1.38) | <0.001 |
| Stroke | | | | | | |
| hsCRP < 1.015 | Reference | | Reference | | Reference | |
| hsCRP ≥ 1.015 | 1.57 (1.30, 1.90) | <0.001 | 1.49 (1.24, 1.81) | <0.001 | 1.38 (1.14, 1.68) | <0.001 |
| Scenario 2 | | | | | | |
| CVD | | | | | | |
| FI ≤ 0.10 | Reference | | Reference | | Reference | |
| 0.10 < FI < 0.25 | 1.61 (1.41, 1.83) | <0.001 | 1.57 (1.37, 1.79) | <0.001 | 1.55 (1.36, 1.77) | <0.001 |
| FI ≥ 0.25 | 2.64 (2.18, 3.21) | <0.001 | 2.51 (2.05, 3.08) | <0.001 | 2.46 (2.01, 3.01) | <0.001 |
| Heart disease | | | | | | |
| FI ≤ 0.10 | Reference | | Reference | | Reference | |
| 0.10 < FI < 0.25 | 1.73 (1.48, 2.02) | <0.001 | 1.70 (1.45, 1.99) | <0.001 | 1.68 (1.43, 1.97) | <0.001 |
| FI ≥ 0.25 | 2.66 (2.11, 3.36) | <0.001 | 2.57 (2.02, 3.27) | <0.001 | 2.52 (1.98, 3.20) | <0.001 |
| Stroke | | | | | | |
| FI ≤ 0.10 | Reference | | Reference | | Reference |  |
| 0.10 < FI < 0.25 | 1.63 (1.31, 2.03) | <0.001 | 1.59 (1.27, 1.99) | <0.001 | 1.58 (1.25, 1.95) | <0.001 |
| FI ≥ 0.25 | 3.19 (2.35, 4.33) | <0.001 | 3.04 (2.21, 4.18) | <0.001 | 2.93 (2.13, 4.04) | <0.001 |

^a^Abbreviations: CVD, cardiovascular disease; hsCRP, high-sensitive C-reactive protein; FI, frailty index; HR, hazard ratios; CI, confidence interval.

^b^Adjusted Model I: age, sex, marital status, educational level, smoking status, drinking status and body mass index were adjusted; Adjusted Model 2: age, sex, marital status, educational level, smoking status, drinking status, body mass index, total cholesterol, high-density lipoprotein cholesterol, low-density lipoprotein cholesterol, glycated hemoglobin, creatinine, creatinine, total triglyceride and fasting blood glucose were adjusted.

^c^Scenario 1: effect of hsCRP on CVD; scenario 2: effect of FI on CVD.

**Supplementary Table 6.** Associations of different groups of FI and hsCRP with heart disease incidence stratified by different factors.

| Subgroup | Group1  (n=1094) | Group2  (n= 868) | Group3  (n=1446) | Group4  (n=1452) | Group5  (n=157) | Group6  (n=222) | P for interaction |
| --- | --- | --- | --- | --- | --- | --- | --- |
|  | HR (95% CI） | HR (95% CI） | HR (95% CI） | HR (95% CI） | HR (95% CI） | HR (95% CI） |  |
| Age | | | | | | | 0.901 |
| <60 | Reference | 1.02 (0.74, 1.42) | 1.55 (1.15, 2.00) | 2.01 (1.53, 2.63) | 2.02 (1.14, 3.57) | 3.13 (1.95, 5.00) |  |
| >=60 | Reference | 1.74 (0.99, 3.05) | 2.71 (1.67, 4.39) | 2.97 (1.85, 4.77) | 3.45 (1.87, 6.37) | 4.87 (2.90, 8.17) |  |
| Gender | | | | | | | 0.879 |
| Female | Reference | 0.98 (0.68, 1.42) | 1.60 (1.20, 2.14) | 1.89 (1.42, 2.52) | 2.28 (1.46, 3.57) | 3.05 (2.07, 4.49) |  |
| Male | Reference | 1.32 (0.71, 2.44) | 1.81 (1.02, 3.25) | 2.28 (1.28, 4.05) | 1.82 (0.73, 4.56) | 3.62 (1.86, 7.04) |  |
| Married | | | | | | | 0.808 |
| No | Reference | 0.96 (0.73, 1.27) | 1.48 (1.18, 1.86) | 1.84 (1.47, 2.30) | 1.97 (1.31, 2.97) | 3.00 (2.19, 4.11) |  |
| Yes | Reference | 0.92 (0.30, 2.83) | 1.26 (0.50, 3.20) | 1.25 (0.50, 3.15) | 1.33 (0.40, 4.39) | 1.62 (0.50, 5.20) |  |
| Smoking status | | | | | | | 0.935 |
| Never | Reference | 0.95 (0.68, 1.34) | 1.54 (1.17, 2.02) | 1.81 (1.38, 2.38) | 1.98 (1.25, 3.14) | 2.66 (1.80, 3.96) |  |
| Current/former | Reference | 1.93 (1.03, 3.61) | 2.66 (1.50, 4.72) | 3.28 (1.86, 5.80) | 3.46 (1.57, 7.66) | 6.16 (3.24, 11.69) |  |
| Drinking status | | | | | | | 0.092 |
| Never | Reference | 0.84 (0.61, 1.17) | 1.45 (1.12, 1.87) | 1.82 (1.42, 2.34) | 2.13 (1.45, 3.20) | 2.68 (1.95, 3.76) |  |
| Current/former | Reference | 2.25 (1.14, 4.46) | 2.69 (1.43, 5.06) | 2.84 (1.49, 5.42) | 1.25 (0.28, 5.59) | 7.00 (2.83, 17.34) |  |
| Dyslipidaemia | | | | | | | 0.670 |
| No | Reference | 1.00 (0.68, 1.46) | 1.36 (0.99, 1.86) | 1.84 (1.35, 2.49) | 1.95 (1.14, 3.35) | 2.38 (1.49, 3.81) |  |
| Yes | Reference | 1.24 (0.68, 2.25) | 2.07 (1.20, 3.59) | 2.24 (1.29, 3.91) | 2.48 (1.22, 5.04) | 4.13 (2.24, 7.61) |  |
| Hypertension | | | | | | | 0.307 |
| No | Reference | 1.33 (0.89, 1.99) | 1.89 (1.36, 2.64) | 2.10 (1.50, 2.94) | 2.06 (1.07, 3.98) | 3.86 (2.42, 6.14) |  |
| Yes | Reference | 1.22 (0.74, 2.11) | 1.98 (1.20, 3.27) | 2.51 (1.53, 4.13) | 2.91 (1.57, 5.39) | 3.64 (2.06, 6.43) |  |
| Kidney disease | | | | | | | 0.732 |
| No | Reference | 0.89 (0.61, 1.30) | 1.37 (1.02, 1.83) | 1.59 (1.19, 2.13) | 1.90 (1.15, 3.12) | 2.37 (1.55, 3.62) |  |
| Yes | Reference | 1.17 (0.68, 2.02) | 1.78 (1.08, 2.94) | 2.20 (1.34, 3.63) | 2.11 (1.05, 4.23) | 3.77 (2.12, 6.70) |  |
| Diabetes | | | | | | | 0.966 |
| No | Reference | 1.01 (0.70, 1.46) | 1.63 (1.21, 2.18) | 1.86 (1.38, 2.51) | 2.25 (1.34, 3.78) | 2.98 (1.93, 4.60) |  |
| Yes | Reference | 1.20 (0.70, 2.05) | 1.70 (1.03, 2.80) | 2.15 (1.31, 3.54) | 2.11 (1.08, 4.13) | 3.48 (1.97, 6.17) |  |

^a^Abbreviations: hsCRP, high-sensitive C-reactive protein; FI, frailty index; HR, hazard ratios; CI, confidence interval.

^b^ This analysis was implemented in the adjusted Model 2: age, sex, marital status, educational level, smoking status, drinking status, body mass index, total cholesterol, high-density lipoprotein cholesterol, low-density lipoprotein cholesterol, glycated hemoglobin, creatinine, creatinine, total triglyceride and fasting blood glucose were adjusted.

^c^Group 1: FI ≤ 0.10 and hsCRP < 1.015 mg/L; Group 2: FI ≤ 0.10 and hsCRP ≥ 1.015 mg/L; Group 3: 0.10 < FI < 0.25 and hsCRP < 1.015 mg/L; Group 4: 0.10 < FI < 0.25 and hsCRP ≥ 1.015 mg/L; Group 5: FI ≥ 0.25 and hsCRP < 1.015 mg/L; Group 6: FI ≥ 0.25 and hsCRP ≥ 1.015 mg/L.

**Supplementary Table 7**. Associations of different groups of FI and hsCRP with stroke incidence stratified by different factors.

| Subgroup | Group1  (n=1094) | Group2  (n= 868) | Group3  (n=1446) | Group4  (n=1452) | Group5  (n=157) | Group6  (n=222) | P for interaction |
| --- | --- | --- | --- | --- | --- | --- | --- |
|  | HR (95% CI） | HR (95% CI） | HR (95% CI） | HR (95% CI） | HR (95% CI） | HR (95% CI） |  |
| Age | | | | | | | 0.456 |
| <60 | Reference | 1.64 (1.00, 2.67) | 1.76 (1.11, 2.78) | 2.60 (1.68, 4.04) | 3.36 (1.52, 7.45) | 6.41 (3.47, 11.85) |  |
| >=60 | Reference | 2.61 (1.17, 5.80) | 3.34 (1.62, 6.86) | 3.72 (1.83, 7.57) | 4.01 (1.62, 9.93) | 7.21 (3.39, 15.35) |  |
| Gender | | | | | | | 0.966 |
| Female | Reference | 1.63 (0.92, 2.90) | 1.78 (1.07, 2.95) | 2.15 (1.30, 3.54) | 2.37 (1.10, 5.08) | 4.67 (2.56, 8.54) |  |
| Male | Reference | 1.72 (0.73, 4.09) | 2.09 (0.91, 4.79) | 2.78 (1.22, 6.35) | 3.89 (1.37, 11.06) | 5.80 (2.37, 14.24) |  |
| Married | | | | | | | 0.576 |
| No | Reference | 1.35 (0.90, 2.00) | 1.66 (1.17, 2.36) | 2.16 (1.51, 3.00) | 2.78 (1.56, 4.95) | 4.71 (3.04, 7.31) |  |
| Yes | Reference | 4.91 (1.39, 17.37) | 3.53 (1.13, 11.02) | 4.48 (1.49, 13.41) | 2.78 (0.51, 14.98) | 6.96 (1.86, 26.05) |  |
| Smoking status | | | | | | | 0.640 |
| Never | Reference | 1.62 (0.93, 2.82) | 2.14 (1.32, 3.48) | 2.57 (1.58, 4.16) | 3.27 (1.60, 6.70) | 6.16 (3.45, 10.98) |  |
| Current/former | Reference | 2.72 (1.09, 6.80) | 2.72 (1.13, 6.56) | 3.75 (1.57, 8.93) | 3.92 (1.28, 12.05) | 6.77 (2.61, 17.55) |  |
| Drinking status | | | | | | | 0.280 |
| Never | Reference | 1.34 (0.85, 2.10) | 1.51 (1.02, 2.24) | 1.73 (1.17, 2.55) | 2.31 (1.27, 4.21) | 3.55 (2.21, 5.72) |  |
| Current/former | Reference | 0.92 (0.34, 2.49) | 1.08 (0.42, 2.79) | 1.83 (0.73, 4.62) | 1.10 (0.22, 5.58) | 4.75 (1.52, 14.80) |  |
| Dyslipidaemia | | | | | | | 0.370 |
| No | Reference | 1.52 (0.86, 2.70) | 1.58 (0.95, 2.64) | 2.70 (1.66, 4.38) | 2.71 (1.23, 5.96) | 5.34 (2.90, 9.85) |  |
| Yes | Reference | 1.86 (0.78, 4.45) | 2.36 (1.04, 5.38) | 2.36 (1.03, 5.41) | 3.06 (1.10, 8.53) | 5.17 (2.11, 12.68) |  |
| Hypertension | | | | | | | 0.303 |
| No | Reference | 0.92 (0.52, 1.60) | 1.35 (0.87, 2.11) | 1.66 (1.07, 2.56) | 1.87 (0.82, 4.26) | 4.47 (2.57, 7.80) |  |
| Yes | Reference | 1.41 (0.67, 2.98) | 1.46 (0.71, 2.99) | 1.89 (0.93, 3.84) | 2.12 (0.87, 5.16) | 3.20 (1.44, 7.10) |  |
| Kidney disease | | | | | | | 0.886 |
| No | Reference | 1.57 (0.87, 2.86) | 1.77 (1.06, 2.97) | 2.28 (1.38, 3.76) | 2.23 (0.97, 5.10) | 5.50 (3.01, 10.06) |  |
| Yes | Reference | 1.61 (0.71, 3.63) | 1.90 (0.87, 4.13) | 2.39 (1.10, 5.18) | 3.34 (1.29, 8.65) | 4.31 (1.82, 10.19) |  |
| Diabetes | | | | | | | 0.409 |
| No | Reference | 1.91 (1.06, 3.45) | 2.02 (1.20, 3.42) | 3.04 (1.86, 5.07) | 3.18 (1.38, 7.32) | 4.90 (2.49, 9.65) |  |
| Yes | Reference | 2.40 (1.07, 5.38) | 2.97 (1.38, 6.45) | 3.36 (1.56, 7.23) | 4.46 (1.76, 11.50) | 8.69 (3.83, 19.73) |  |

^a^Abbreviations: hsCRP, high-sensitive C-reactive protein; FI, frailty index; HR, hazard ratios; CI, confidence interval.

^b^ This analysis was implemented in the adjusted Model 2: age, sex, marital status, educational level, smoking status, drinking status, body mass index, total cholesterol, high-density lipoprotein cholesterol, low-density lipoprotein cholesterol, glycated hemoglobin, creatinine, creatinine, total triglyceride and fasting blood glucose were adjusted.

^c^Group 1: FI ≤ 0.10 and hsCRP < 1.015 mg/L; Group 2: FI ≤ 0.10 and hsCRP ≥ 1.015 mg/L; Group 3: 0.10 < FI < 0.25 and hsCRP < 1.015 mg/L; Group 4: 0.10 < FI < 0.25 and hsCRP ≥ 1.015 mg/L; Group 5: FI ≥ 0.25 and hsCRP < 1.015 mg/L; Group 6: FI ≥ 0.25 and hsCRP ≥ 1.015 mg/L


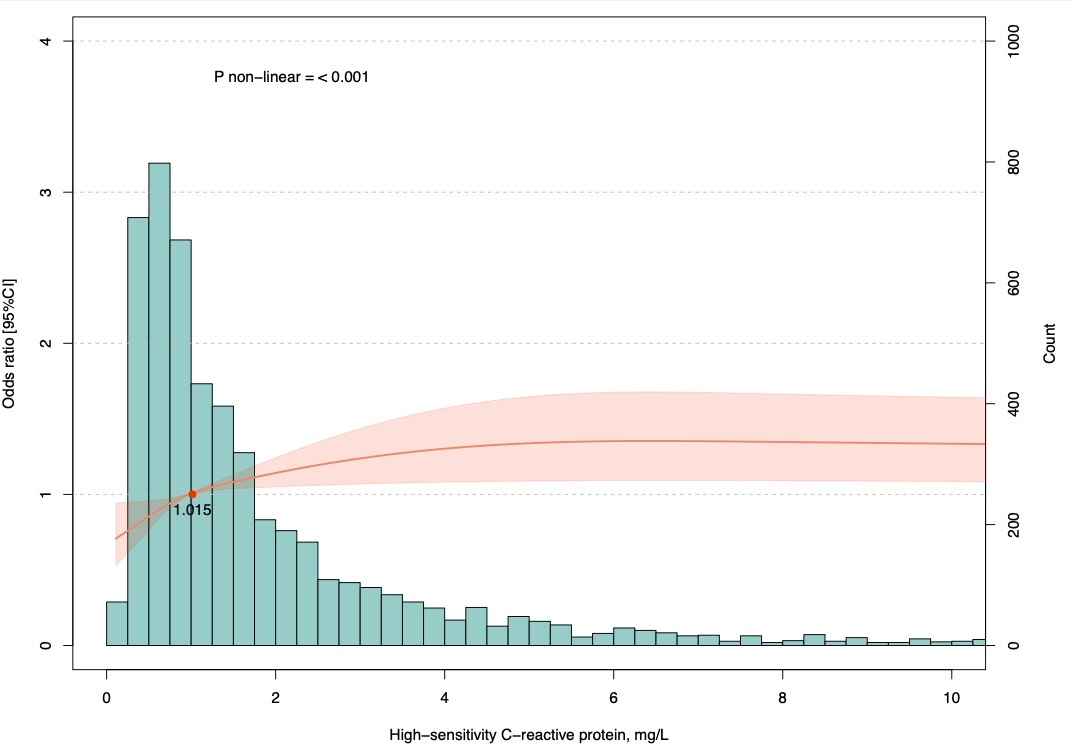


**Supplementary Figure 1.** Nonlinear association between high-sensitive C-reactive protein and cardiovascular disease


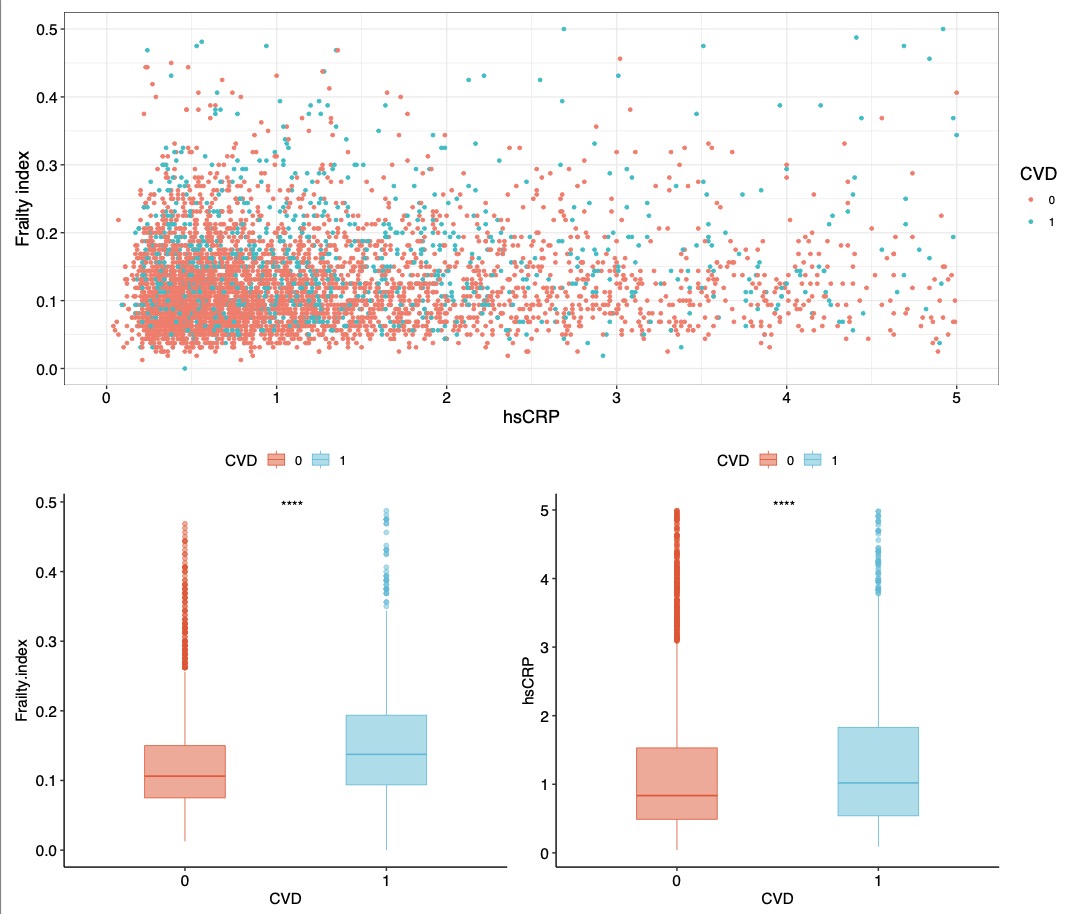


**Supplementary Figure 2.** The codistribution of the frailty index and high-sensitive C-reactive protein stratified by incident cardiovascular disease.


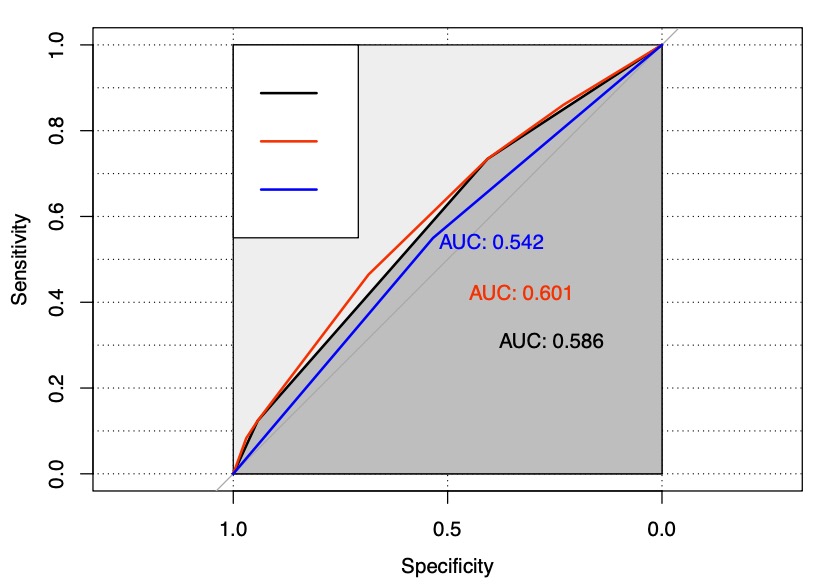


**Supplementary Figure 3.** The Receiver operating characteristic curves of high sensitive C-reactive protein, frailty index and their combination about new-onset cardiovascular disease. Red line for the combination. Black line for frailty index. Blue line for high sensitive C-reactive protein. The area under the curve of the combination is significantly higher than that of high sensitive C-reactive protein or frailty index (p < 0.05).


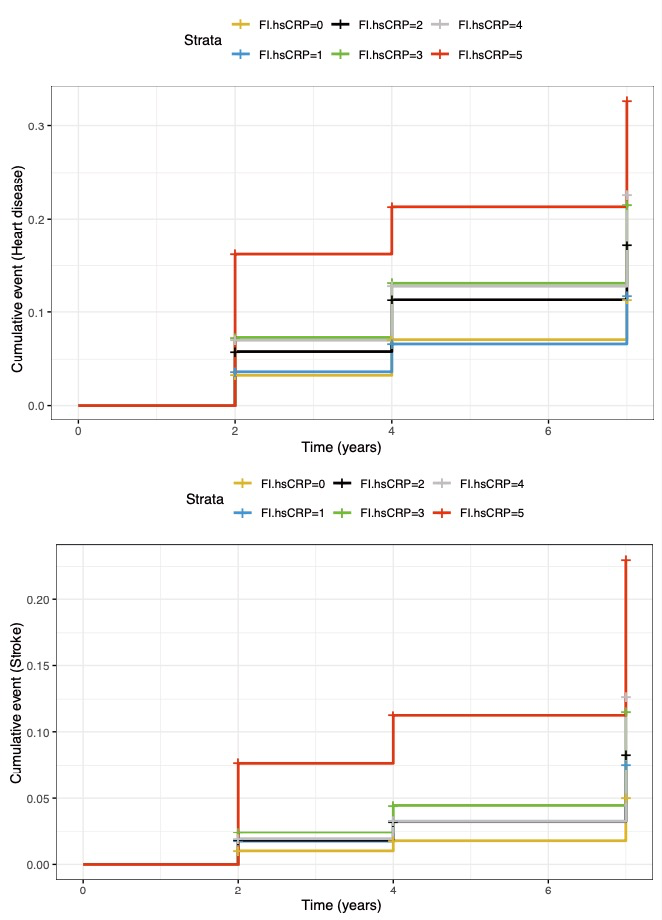


**Supplementary Figure 4.** The Kaplan–Meier curves of the cumulative incidence of heart disease and stroke. FI.hsCRP = 0: FI ≤ 0.10 and hsCRP < 1.015 mg/L; FI.hsCRP = 1: FI ≤ 0.10 and hsCRP ≥ 1.015 mg/L; FI.hsCRP = 2: 0.10 < FI < 0.25 and hsCRP < 1.015 mg/L; FI.hsCRP = 3: 0.10 < FI < 0.25 and hsCRP ≥ 1.015 mg/L; FI.hsCRP = 4: FI ≥ 0.25 and hsCRP < 1.015 mg/L; FI.hsCRP = 5: FI ≥ 0.25 and hsCRP ≥ 1.015 mg/L.


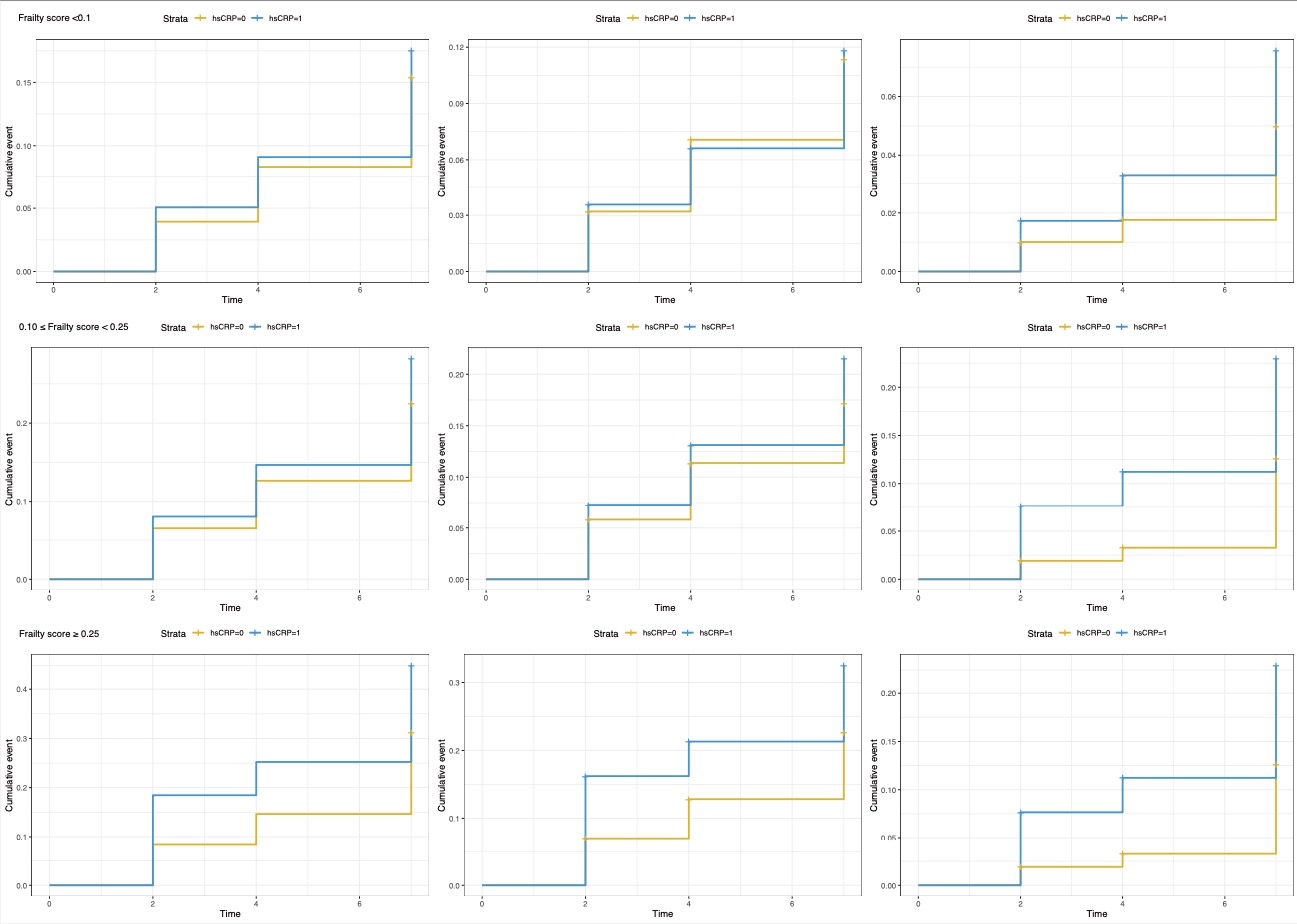


**Supplementary Figure 5.** The Kaplan–Meier curves of the cumulative incidence of cardiovascular disease stratified by frailty index. hsCRP = 0: hsCRP < 1.015 mg/L; hsCRP = 1: hsCRP ≥ 1.015 mg/L.


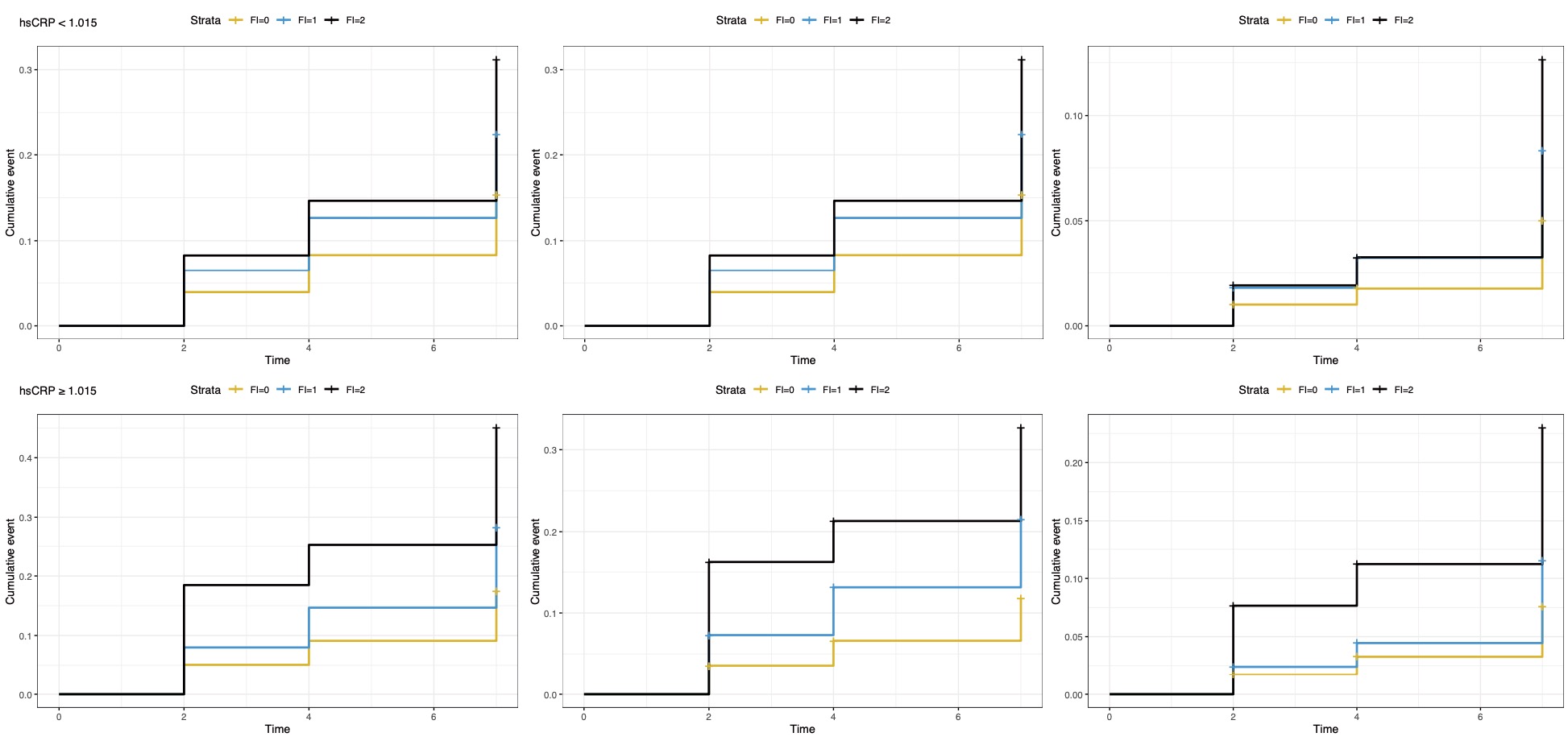


**Supplementary Figure 6.** The Kaplan–Meier curves of the cumulative incidence of cardiovascular disease stratified by high sensitive C-reactive protern. FI = 0: FI ≤ 0.10; FI=1: 0.10 < FI < 0.25; FI = 2: FI ≥ 0.25.


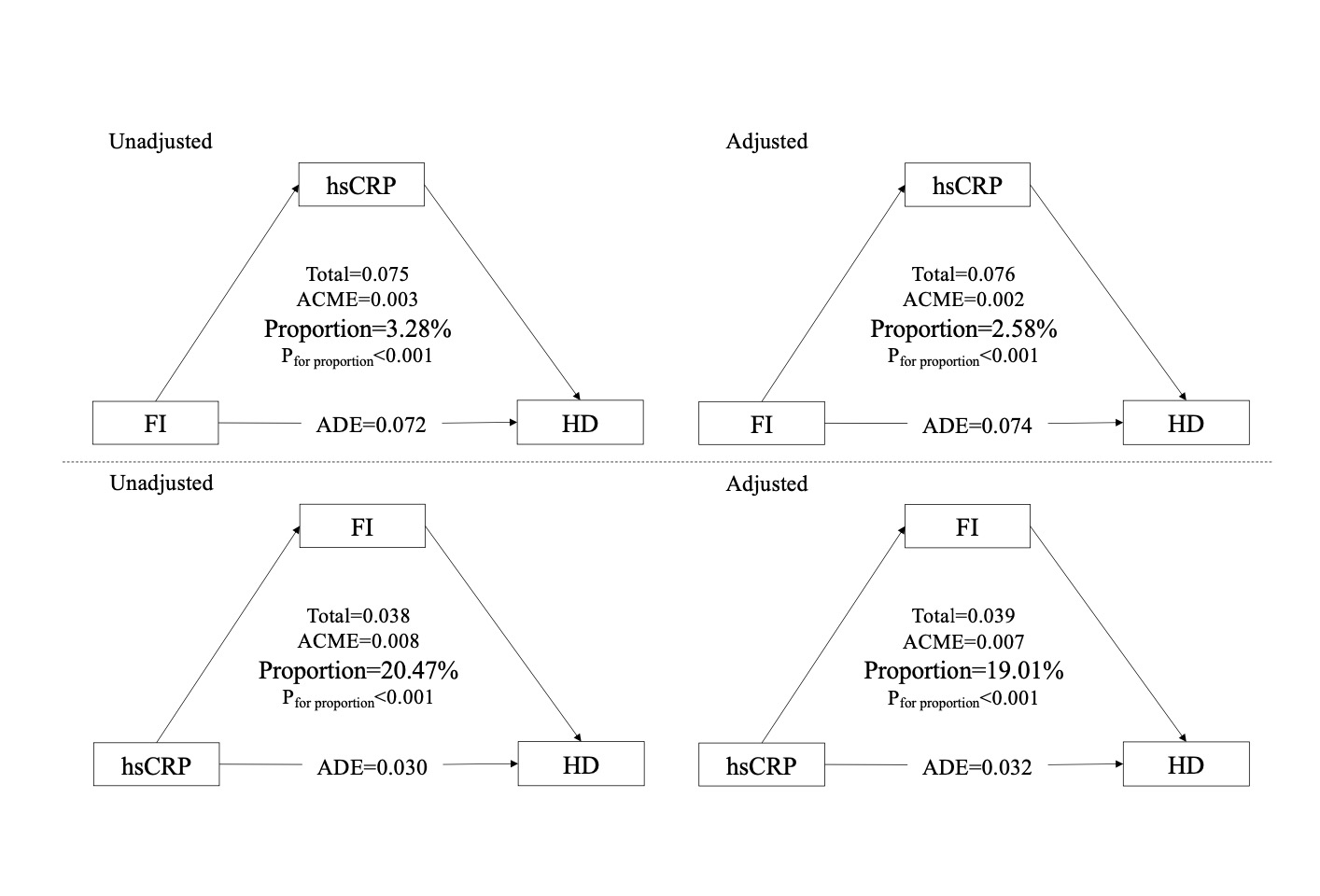


**Supplementary Figure 7.** Mutual mediation effects of the high sensitive C-reactive protein and frailty index on heart disease. Abbreviations: HD, heart disease; hsCRP, high-sensitive C-reactive protein; FI, frailty index.


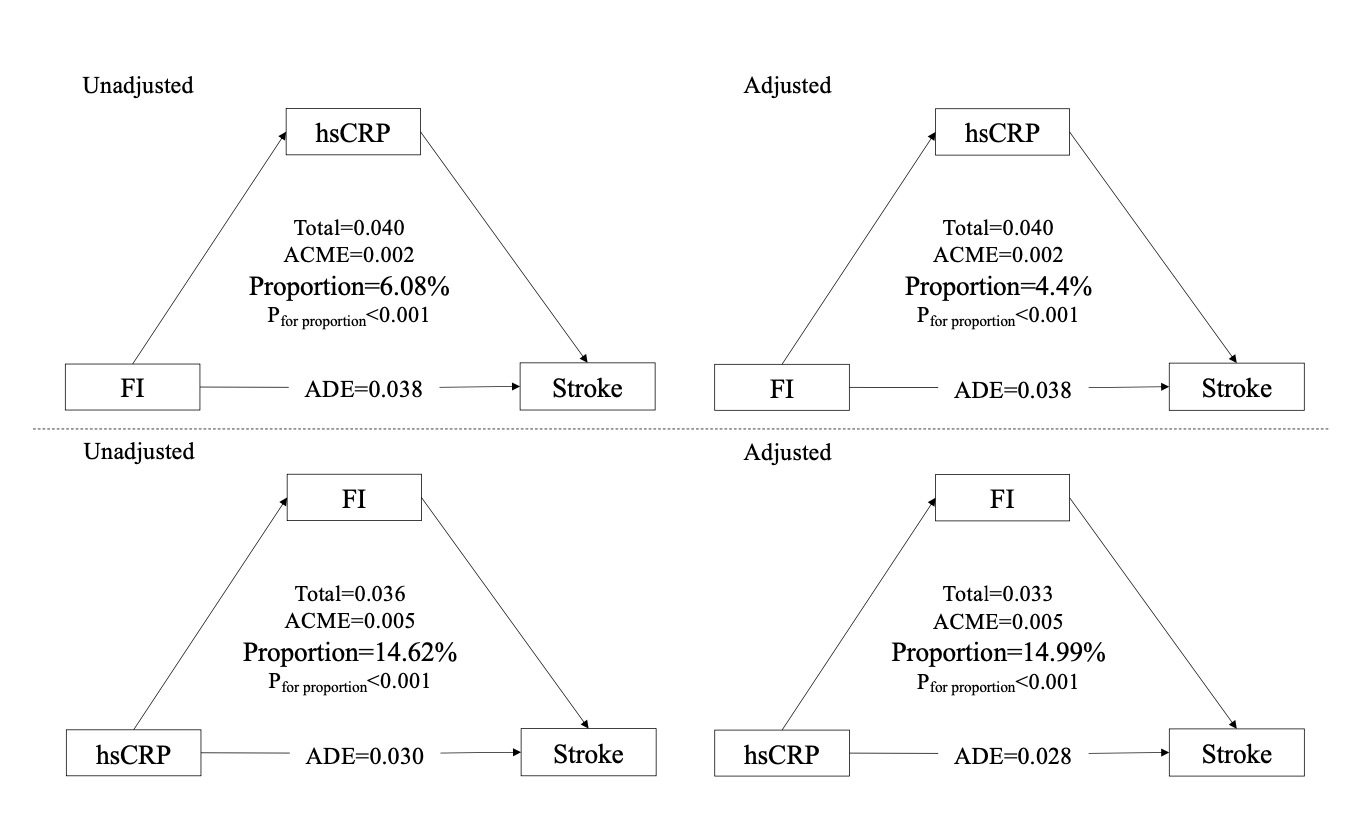


**Supplementary Figure 8.** Mutual mediation effects of the high sensitive C-reactive protein and frailty index on stroke. Abbreviations: hsCRP, high-sensitive C-reactive protein; FI, frailty index.
